# Arginine-Restricted Therapy Resistant Bilateral Macular Edema Associated with Gyrate Atrophy

**DOI:** 10.1155/2015/137270

**Published:** 2015-12-06

**Authors:** Sibel Doguizi, Mehmet Ali Sekeroglu, Mustafa Alpaslan Anayol, Pelin Yilmazbas

**Affiliations:** Ulucanlar Eye Training and Research Hospital, Department of Ophthalmology, 06240 Ankara, Turkey

## Abstract

*Introduction*. Gyrate atrophy is a rare genetical metabolic disorder affecting vision. Here, we report a 9-year-old boy with gyrate atrophy associated with bilateral macular edema at the time of diagnosis and the effect of long term metabolic control on macular edema.* Case Presentation*. A 9-year-old boy presented with a complaint of low visual acuity (best corrected visual acuity: 20/80 in both eyes, refractive error: −12.00 D). Dilated fundus examination revealed multiple bilateral, sharply defined, and scalloped chorioretinal atrophy areas in the midperipheral and peripheral zone. Spectral-domain optical coherence tomography revealed bilateral cystoid macular edema in both eyes. Serum ornithine level was high (622 *μ*mol/L). An arginine-restricted diet reduced serum ornithine level (55 *μ*mol/L). However, visual findings including macular edema remained unchanged in 2 years of follow-up.* Conclusion*. Arginine-restricted diet did not improve macular edema in our patient with gyrate atrophy. A more comprehensive understanding of the underlying factors for macular edema will lead to the development of effective therapies.

## 1. Introduction

Gyrate atrophy is a rare, genetically determined, autosomal recessive, metabolic disorder associated with increased plasma ornithine, caused by the deficiency of the vitamin B6-dependent enzyme ornithine ketoacid aminotransferase [1]. Patients typically report night blindness, loss of peripheral vision, or low visual acuity in the first or second decade of life, and these complaints are accompanied by the appearance of sharply demarcated circular areas of chorioretinal atrophy. As it is a progressive chorioretinal degenerative disorder, the chorioretinal lesions increase in size and number with increasing age. The macula is apparently the most resistant to disease progression [2]. Myopia [2], early posterior subcapsular cataract [2], choroidal neovascularization [3], cystoid macular edema [4], epiretinal membrane [4], and macular hole [5] may also accompany chorioretinal atrophic lesions. In this report, we describe a child with gyrate atrophy associated with bilateral macular edema at the time of diagnosis, which did not improve within 2 years even after strict metabolic control of the disease.

## 2. Case Presentation

**Figure 1:**
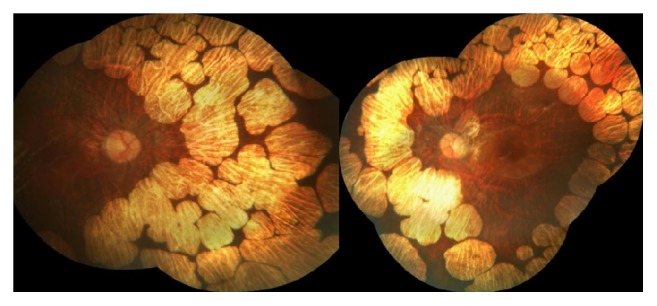
Fundus photography of gyrate atrophy.

**Figure 2:**
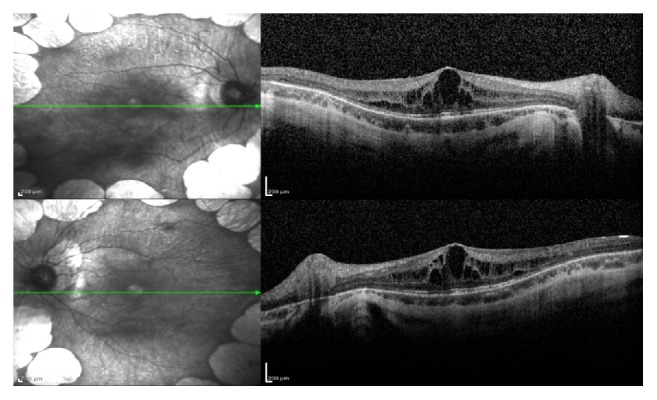
At the time of diagnosis, SD OCT revealed bilateral macular edema in both eyes.

**Figure 3:**
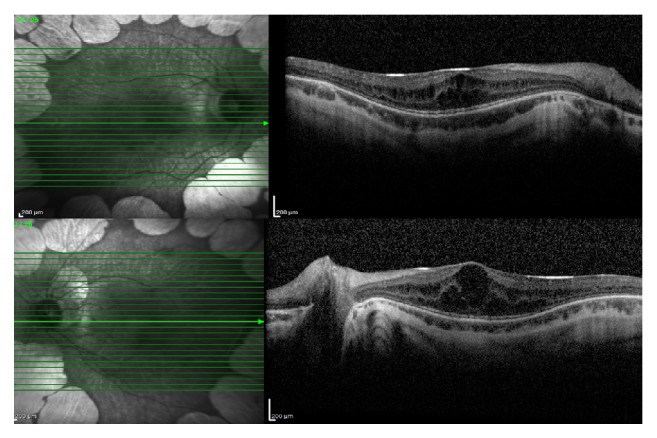
After two years, SD OCT revealed bilateral macular edema; SD OCT findings remained unchanged.

A 9-year-old boy presented with a complaint of low visual acuity. Best corrected visual acuity (BCVA) was 20/80 in both eyes, with a refractive error of −12.00 D. Anterior segment examinations were normal bilaterally. Dilated fundus examination revealed multiple bilateral, sharply defined, and scalloped chorioretinal atrophy areas in the midperipheral and peripheral zone (Figure 1). Spectral-domain optical coherence tomography (SD OCT) revealed bilateral cystoid macular edema in both eyes (Figure 2). With these findings, the gyrate atrophy is the probable diagnosis. Routine blood tests were normal, but amino acid analysis revealed a high serum ornithine level (622 *μ*mol/L), which helps to make the definite diagnosis. In addition, ornithine level was high in the urine analysis (234 nmol/mg creatinine). Also all family members were examined and no similar findings were found (parents and two sisters). The patient consulted with a pediatric metabolic disease specialist. After starting to consume an arginine-restricted diet, serum ornithine level reduced within two months (55 *μ*mol/L). His serum ornithine concentrations were well controlled during the last 22 months over 2-year follow-up. However, his BCVA, fundus appearance, and macular edema remained unchanged in 2 years of follow-up (Figure 3).

## 3. Discussion

Gyrate atrophy is a progressive disorder, and the macular area is relatively spared until late phases of the disease process [2]. Feldman et al. [4] reported a patient with an epiretinal membrane and cystoid macular edema associated with gyrate atrophy, and Oliveira et al. [6] determined cystoid macular edema in a 12-year-old boy with gyrate atrophy. In addition, from a series of 21 patients with gyrate atrophy, Vannas-Sulonen [7] reported one patient (5%) with bilateral cystoid macular edema and confirmed using fundus fluorescein angiogram. After the widespread use of SD OCT in retinal disorders, previously unrecognized aspects of some retinal disorders started to be recognized. Sergouniotis et al. [8] detected the signs of multiple intraretinal cysts in 5 out of 7 gyrate atrophy patients on the SD OCT, and thickening was evident in the fovea of younger patients, despite the posterior pole appearing relatively preserved. They reported that macular edema is a common finding, and the fovea is relatively thick in early stages of disease [8]. Katagiri et al. also reported two brothers with long term follow-up of macular edema by arginine-restricted therapy and determined that macular edema did not improve. Similarly we followed up our patient who was on strict arginine-restricted diet for 2 years and determined that macular edema remained unchanged during the follow-up [9].

Various pathogenetic hypotheses have been offered to explain the cystic macular lesions in retinal dystrophies [10–12]. Increased blood-retinal barrier permeability, tangential vitreous traction, mutations in the retinoschisin gene, and reduction of retina pigment epithelium pumping mechanism have been described. In gyrate atrophy, impairment of the blood-retinal barrier is the most likely mechanism, and it is possible that this involves an underlying autoimmune process [13]. In the light of this hypothesis, Vasconcelos-Santos et al. [14] reported a patient with gyrate atrophy and macular edema, which was treated with intravitreal triamcinolone injection, obtaining only temporary reduction in macular edema. In our patient, we did not find enough reason to use triamcinolone or vascular endothelial growth factor inhibitors because the underlying pathogenesis of the edema was still unclear and an effective treatment was not defined yet.

In conclusion, macular edema is an important finding in patients with gyrate atrophy. SD OCT examination is critical for diagnosing macular edema, even with a clinically normal appearing macula, and for the follow-up. Arginine-restricted diet helped to lower serum ornithine levels but did not improve macular edema. The mechanism of the edema is still unclear, and a more comprehensive understanding of the underlying factors will lead to the development of effective therapies.
